# Biopsy confirmation of metastatic sites in breast cancer patients: clinical impact and future perspectives

**DOI:** 10.1186/bcr3630

**Published:** 2014-03-21

**Authors:** Carmen Criscitiello, Fabrice André, Alastair M Thompson, Michele De Laurentiis, Angela Esposito, Lucia Gelao, Luca Fumagalli, Marzia Locatelli, Ida Minchella, Franco Orsi, Aron Goldhirsch, Giuseppe Curigliano

**Affiliations:** 1 Division of Early Drug Development for Innovative Therapies, Istituto Europeo di Oncologia, Via Ripamonti 435, 20133 Milano, Italia; 2 INSERM Unit U981, Gustave Roussy Institut, Villejuif 94805, France; 3 Department of Medical Oncology, Gustave Roussy Institute, Villejuif 94805, France; 4 Dundee Cancer Centre, Ninewells Hospital and Medical School, University of Dundee, Clinical Research Centre Building, Dundee DD1 9SY, UK; 5 Department of Breast Oncology, Istituto nazionale Tumori ‘Fondazione Pascale’, via Mariano Semmola, 80131 Napoli, Italia; 6 Unit of Interventional Radiology, Istituto Europeo di Oncologia, Via Ripamonti 435, 20133 Milano, Italia

## Abstract

Determination of hormone receptor (estrogen receptor and progesterone receptor) and human epidermal growth factor receptor 2 status in the primary tumor is clinically relevant to define breast cancer subtypes, clinical outcome, and the choice of therapy. Retrospective and prospective studies suggest that there is substantial discordance in receptor status between primary and recurrent breast cancer. Despite this evidence and current recommendations, the acquisition of tissue from metastatic deposits is not routine practice. As a consequence, therapeutic decisions for treatment in the metastatic setting are based on the features of the primary tumor. Reasons for this attitude include the invasiveness of the procedure and the unreliable outcome of biopsy, in particular for biopsies of lesions at complex visceral sites. Improvements in interventional radiology techniques mean that most metastatic sites are now accessible by minimally invasive methods, including surgery. In our opinion, since biopsies are diagnostic and changes in biological features between the primary and secondary tumors can occur, the routine biopsy of metastatic disease needs to be performed. In this review, we discuss the rationale for biopsy of suspected breast cancer metastases, review issues and caveats surrounding discordance of biomarker status between primary and metastatic tumors, and provide insights for deciding when to perform biopsy of suspected metastases and which one (s) to biopsy. We also speculate on the future translational implications for biopsy of suspected metastatic lesions in the context of clinical trials and the establishment of bio-banks of biopsy material taken from metastatic sites. We believe that such bio-banks will be important for exploring mechanisms of metastasis. In the future, advances in targeted therapy will depend on the availability of metastatic tissue.

## Introduction

Confirmatory biopsy of breast cancer metastases may have many potential benefits: confirming metastatic disease; disclosing non-malignant disease or other primary tumors; and confirming concordance (or discordance) of biological features of disease such as estrogen receptor (ER), progesterone receptor (PgR) and human epidermal growth factor receptor (HER)2 status. Any one of these may contribute to the optimal management of patients with metastatic breast cancer. Discordance in ER, PgR and HER2 status between primary and metastatic breast cancer has been frequently reported [1]. Although evidence for this has come mostly from retrospective analyses, a few more recent studies have prospectively evaluated the impact of phenotype discordance in patient management (for example, treatment planning) and survival [2]. Re-evaluating the biological features of disease using metastatic lesions has been largely individualized, although recent practice guidelines of the National Comprehensive Cancer Network recommend biopsy of metastatic sites, especially when they represent the first recurrence of disease and/or ER/PgR/HER2 status is unknown or was originally negative. However, the acquisition of tissue from suspected breast cancer metastases is not always performed in routine practice. Therefore, therapeutic decisions in the metastatic setting are often based on the features of the primary tumor. This often adopted attitude in routine clinical practice is contrary to increasing evidence on the extent and prevalence of intra-tumor heterogeneity from advanced sequencing technologies, which have provided insight into the remarkable genetic complexity of cancers [3]. Intra-tumor heterogeneity refers to the existence of subpopulations of cells within a primary tumor and its metastases that have distinct genotypes and phenotypes and may have different biological behaviors [3]. Intra-tumor heterogeneity has implications for cancer therapeutics and biomarker discovery, particularly in the era of personalized medicine, thus ultimately impacting on clinical outcome. In order to better understand the processes that cause intra-tumor heterogeneity and improve patient care and clinical outcomes, clinical trials including comprehensive tissue collection protocols are warranted.

## Potential benefits and rationale for biopsying metastatic sites

Biopsies of breast cancer metastases have many potential benefits. Primarily, biopsying the metastatic site can establish a diagnosis in patients with a single metastasis who were until that time not known to have advanced disease. Biopsies of suspected metastatic lesions can also reveal an unsuspected non-malignant process or other primary cancer. Quite recently, two independent, prospective studies reported on the clinical impact of biopsy of recurrent lesions [4,5]. The single-centre Canadian DESTINY study and the multicentre UK Breast Recurrence In Tissues Study (BRITS) revealed non-malignant processes in 3 out of 121 (2.5%) and in 18 out of 205 (8.8%) patients with suspected metastases, respectively [4,5]. One woman who participated in the DESTINY study had a second malignancy (0.8%) [4]. Last but not least, biopsies can corroborate or refute expression of breast cancer-related biomarkers that influence treatment choice.

One of the main reasons supporting the need for biopsies of breast cancer metastases lies in the fact that tissue sampling may be helpful in individualizing therapy according to the profile of metastatic disease rather than the primary tumor. The discordance in ER, PgR and HER2 receptor status between primary and metastatic breast cancer has been constantly reported [1] (Table 1). This evidence has been mostly derived from retrospective analyses investigating ER, PgR and HER2 status in heterogeneous sites of relapse [1]. Recently, a meta-analysis showed that the rates of discordance for ER, PgR and HER2 status were 20%, 33% and 8%, respectively [6].

**Table 1:** Data on discordance of ER, PgR and HER2 status between primary and metastatic breast cancer

**Reference**	**ER discordance (%)**	**PgR discordance (%)**	**HER2 discordance (%)**
Lindstrom *et al*. [7]	32.4	40.7	14.5
Niikura *et al*. [8]	NR	NR	43
Curigliano *et al*. [9]	15	49	14
Gong *et al*. [10]	7	NR	NR
Amir *et al*. [2]	12.6	31.2	5.5
Bogina *et al*. [11]	6	21	1
Wilking *et al*. [12]	NR	NR	10
Aitken *et al*. [13]	28	23	9
Amir *et al*. [4]	16	40.4	9.6
Idirisinghe *et al*. [14]	16	38	5
Simmons *et al*. [15]	40	40	8
Liedtke *et al*. [16]	18	40	14
Lower *et al*. [17]	NR	NR	33
Broom *et al*. [18]	17.7	37.3	5.5
Santinelli *et al*. [19]	NR	NR	18.9
Tapia *et al*. [20]	NR	NR	8
Lower *et al*. [21]	30	39	NR
Carlsson *et al*. [22]	NR	NR	0
Edgerton *et al*. [23]	NR	NR	15
Gancberg *et al*. [24]	NR	NR	9
Simon *et al*. [25]	NR	NR	3
Mobbs *et al*. [26]	14	28	NR
Guarneri *et al*. [27]	22	36	16
Hoefnagel *et al*. [28]	10.3	30	5.2
Thompson *et al*. [5]	10.2	24.8	2.9
Brogi *et al*. [29]	16.2	20.6	5
Chang *et al*. [30]	25	NR	12.5

ER, estrogen receptor; HER, human epidermal growth factor receptor; NR, not reported; PgR, progesterone receptor.

Two alternative explanations may justify this observation: technical issues, including poor reproducibility of the immunohistochemical technique, or actual tumor heterogeneity. The lack of perfect reproducibility in the assessment of ER, PgR and HER2 status has been described in prospective trials based on central pathology review [31,32], and a mathematical model has been proposed foreseeing a discordance rate of at least 10% even using an ideal test yielding 95% accuracy, sensitivity and specificity, a scenario reasonably far from clinical practice, where additional variables may further affect reproducibility [33]. Very likely the technical issue on its own does not completely explain the discrepancy in ER, PgR and HER2 status between primary tumors and relapses, as we would likely observe roughly the same discordance rates for ER, PgR and HER2 status, which is not the case [6]. Moreover, the conversion to negative receptor status is, on average, higher than the positive conversion (24% versus 14% for ER, 46% versus 15% for PgR, and 13% versus 5% for HER2); these would be expected to be similar if occurring by chance or for technical reasons [6]. This observation might be explained by the occurrence of mechanisms of resistance, perhaps as the result of selection fostering ER-, PgR- and HER2-negative tumor clones in the metastases. On the other hand, recent studies based on next-generation sequencing have shed new light on tumor heterogeneity, reinforcing the hypothesis that variation in ER, PgR and HER2 status may actually reflect clonal genome evolution. Tumor heterogeneity may be attributable to tumor biological drift, selective pressures of therapy leading to clonal selection with development of a novel tumor cell clone, or presence of small subclones routinely undetected within the primary tumor [3].

In the era of personalized medicine, biopsies may also improve selection of patients for targeted therapy trials. There is a considerable lack of understanding of metastasis biology due to the imbalance between the abundance of tissue from primary tumors and no cultures of metastasis biopsies. This has led to the assumption that lessons from primary breast cancer extend to metastatic breast cancer. Despite the large number of papers published on the role of genome sequencing in breast cancer, just two [34,35] have focused on the metastatic site, while all the others have focused on primary breast cancer. Communally, 900 primary breast cancers have been sequenced versus 10 breast cancer metastases. The characterization of metastatic breast cancer has been systematically neglected because of the difficulties culturing metastasis biopsies. Hence, it is important to change attitudes in order to better understand how metastases determine outcome in breast cancer. Potentially, we can detect substantial genomic evolution between a primary tumor and its metastases. All existing methods of classifying breast cancer are predicated on primary breast cancer. However, many metastatic-specific mutations do exist, many of which may be driver mutations that could be targeted by drugs, but unless biopsy of metastasis is performed we will not be able to identify these relevant molecular targets.

## Safety of the procedure and logistical considerations

Biopsy cannot always be performed safely for the patient. Generally, biopsy of metastases in patients with breast cancer is carried out by percutaneous image-guided biopsy, which is safe, effective, and is associated with minimal complications and patient discomfort. The Society of Interventional Radiology reports a threshold of 2% for major complications [36]. Generic complications include bleeding, infection, perforation, and unintended organ injury. Moreover, organ-specific complications associated with biopsy can occur, such as hematuria with kidney biopsy or pneumothorax with lung biopsy. Tumor seeding after biopsy is very rare [37]. At the 2013 ASCO annual meeting, Dr André reported that biopsy was complicated by a serious adverse event in 9 out of 423 patients within the SAFIR01 trial [38]. The acceptance, feasibility and safety of sequential biopsies have been studied in the field of clinical trials [39]. Additionally, iterative biopsies may lead to delay in treatment in patients with metastatic breast cancer [4]. Finally, it has to be acknowledged that this procedure is financially expensive.

## Treatment changes according to biopsy of the metastasis

Few studies have reported treatment changes based on analysis of metastases (Table 2). In a pooled analysis of the prospective BRITS and DESTINY studies, biopsy results altered management in 14.2% of cases but the clinical impact is unknown [2]. Modification of therapy was more common when there was gain of receptor expression. Gain of HER2 amplification resulted in a change of therapy in 11 patients, and gain of hormone receptor expression resulted in a change of therapy in 9 patients. Loss of HER2 amplification led to a change in therapy in 1 patient, and loss of hormone receptor expression led to a change of therapy in 11 patients [2]. Overall, biomarker evaluation of metastasis within prospective clinical trials led to therapeutic changes in 17 to 20% of patients [4,5,15]. Loss or gain of biomarker expression between primary tumor and metastasis may not have the same therapeutic impact. Changing therapy based on loss of a biomarker could avoid the prescription of an unnecessary treatment with potential toxicity for the patient, but data are not robust enough to affirm that an agent should be discontinued based on a new biopsy of the metastasis. Conversely, gain of a biomarker could offer the possibility to adopt new therapies but this event is relatively rare. Importantly, we have to highlight that the impact on survival of biopsy-driven treatment has not been reported to date in a prospective randomized trial.

**Table 2:** Change in management based on biopsy

**Reference**	**Number of patients**	**Therapy changed (%)**
Lindstrom *et al*. [7]	1,010	23 (HER2) 50 (ER)
Curigliano *et al*. [9]	255	12.1
Amir *et al*. [2]	289	14.2
Bogina *et al*. [11]	140	7.3
Amir *et al*. [4]	121	14
Simmons *et al*. [15]	40	20
Thompson *et al*. [5]	137	17.5

ER, estrogen receptor; HER2, human epidermal growth factor receptor 2.

## Which metastasis to biopsy and when?

Breast cancer metastases have been poorly studied systematically, with usually only one of a breast cancer patient's metastases biopsied with small tissue sample (s). Whether the capacity to metastasize is an intrinsic property of the tumor or an acquired feature, and whether all cells in a primary tumor are capable of metastasis or just a small subset (subclone) are unknown. It is debatable whether different metastases in the same patient are biologically different, perhaps as a result of selection pressures. To address this issue, autopsy data prospectively comparing primary tumor to paired multiple metastatic sites from eight patients found noticeable similarities in terms of gene expression profiles, suggesting that metastatic capability was an intrinsic property of the primary tumor and not based on clonal selection [40]. Another study compared the expression of biomarkers between primary tumors and their paired metastases and among different metastases from the same patient; this study used archived primary breast cancer and multiple different metastases collected at autopsy from 10 consenting patients [41]. It revealed consistency in ER, PgR and HER2 expression in different metastases even if this was different to the expression in the synchronous primary cancer [41].

Even assuming clinical and genomic heterogeneity across metastases [3], we cannot sample all lesions, clones or genomes in a single patient, as the cost-benefit ratio would be too high. When proposing a biopsy to a patient, we need to consider patient risk, inconvenience and discomfort, although it has been reported that 51% of patients would altruistically consider having biopsies for research purposes only with no clinical implications, even knowing the risks, and 72% would consider having additional biopsies to provide more material for research [42]. Also, there are fiscal implications related to sampling and analysis, although the economic burden of metastatic breast cancer is overall remarkable in terms of monetary cost [43].

Pragmatically, a valid strategy could consist of biopsying and analyzing the first metastasis and, accordingly, selecting the subsequent treatment. Thereafter, for resistant or relapsing lesions, further biopsy may be considered. Where cancer cells cannot be eliminated with a single course of therapy, several steps may be required considering individual clones and subclones within the tumor. The timing of tumor reappearance should also be considered as a variable to support biopsy of metastasis. For late recurrences of ER + breast cancers, the ‘tumor dormancy’ scenario, there is a medical need for confirmatory biopsy to ascertain histology.

## Acquired resistance and biopsy of metastases

Treatment exposure may change the molecular profile of a tumor and this can be assessed by performing biopsy of the metastatic site. Challenges to targeted therapies include acquired and primary resistance. Acquired resistance may develop in most patients with metastatic breast cancer [44]. Some mechanisms by which a tumor stops responding to a given therapy that it had initially responded to have been identified in HER2+ tumors. These include loss of expression of the target as a result of continuous therapy [45], activation of mutations downstream of the target itself, and activation of additional mechanisms that promote cell proliferation [46]. Primary resistance may occur due to lack of target dependency. Furthermore, activation of compensatory pathways may rescue cells from the inhibitory effects of blocking just one target or pathway.

While treatment of HER2+ breast cancer with the HER2-targeted monoclonal antibody trastuzumab has demonstrated significant improvements in patient outcome, nearly 70% of patients with HER2+ breast cancer demonstrate resistance to trastuzumab in the metastatic setting [47-49]. Several mechanisms have been implicated in resistance to trastuzumab, including increased signaling mediated by growth factor receptors, alterations of the HER2 receptor, phosphatase and tensin homolog (PTEN) loss, and aberrant activity of the phosphatidylinositide 3-kinase (PI3K)/mammalian target of rapamycin (mTOR) pathway. Quite recently, contrary to some preclinical and a few clinical studies suggesting a decrease in trastuzumab sensitivity in patients with PTEN- tumors, Perez and colleagues [50] showed that HER2+ breast cancer patients benefit from adjuvant trastuzumab regardless of tumor PTEN status. Therefore, inhibition of the PI3K/Akt/mTOR pathway with a mTOR inhibitor such as everolimus might be a reasonable approach to overcome resistance and restore sensitivity to trastuzumab-based therapy. This hypothesis is supported by preclinical studies in which PI3K inhibitors overcame PTEN loss-induced trastuzumab resistance and slowed the growth of HER2+ breast cancer cells *in vitro* and *in vivo*[51,52]. Such a therapeutic approach has been adopted in the BOLERO-3 (Breast Cancer Trials of Oral Everolimus) clinical trial, in which the addition of everolimus to trastuzumab and vinorelbine significantly prolonged progression-free survival in patients with HER2+ metastatic breast cancer resistant to trastuzumab, as presented by Dr O’Regan during the 2013 ASCO annual meeting (unpublished observations, Ruth O’Regan).

Similarly, mutation frequencies in the ER gene (*ESR1*) differ between primary and metastatic tumors, as seen through large-scale next-generation sequencing of postmenopausal women with ER + metastatic breast cancer treated with everolimus in combination with exemestane within the BOLERO-2 trial, as presented by Dr Piccart at the 2013 IMPAKT meeting (unpublished observations, Martine Piccart). The BOLERO-2-based dataset indicated enrichment in *ESR1* gene mutations from primary tumor samples (7%) compared with metastatic tumor samples (19%). These mutations appear to cluster in the ligand-binding domain, so are probably affecting the binding of estrogen, rendering these tumors likely estrogen-independent.

In such a context, the role of biopsy is crucial to develop drugs that aim at reversing resistance, based on protein assessment of the metastasis. This concept is in contrast to the current concept of ‘oncogene de-addiction’, according to which the oncogenic targets need to be present on the primary tumor. Thus, on one hand the biopsy of the primary tumor may be helpful to develop therapies that aim to de-addict to oncogenes, and on the other biopsy of metastases may be helpful to develop drugs that aim at reversing resistance.

## The role of biopsy for research purposes

Current molecular oncology technology enables us to characterize the complete mutational landscape of cancers [53,54]. This holds great promise with regard to understanding driving genetic aberrations, discovering new therapeutic targets, elucidating tumor genetic heterogeneity and ultimately improving outcomes for cancer patients. Recent studies using massively parallel sequencing have uncovered a large number of potential ‘driver’ mutations that occur at a low frequency but are potentially targetable by agents that are currently available for other clinical indications or for possible breast cancer clinical trials [53,54].

Breast cancer metastases often acquire new molecular aberrations, and different treatment-resistant clones may emerge over time. While the clinical relevance of these phenomena is not yet well understood, it suggests that metastatic lesions should be biopsied to recover the genomic portrait of advanced breast cancers, and that treatment decisions could conceivably be based on these molecular profiles, rather than those of the primary tumors. To this end, the Breast International Group (BIG) is constructing a molecular screening program called PRISM-BC that will facilitate the large-scale screening and sequencing of metastatic breast cancer patients, using metastatic biopsies, to determine which patients would be eligible for phase II downstream therapeutic clinical trials conducted under the BIG umbrella using novel targeted agents. In this context, the French SAFIR02 program (NCT01414933) aims at demonstrating the medical benefits of treatment based on genomic analysis. Only a small proportion of these patients will be identified with putative ‘actionable’ mutations, with the vast majority having ‘non-actionable’ mutations; BIG is committed to building an important research program based on the follow-up of screened but trial-ineligible patients to advance our knowledge of metastatic breast cancer.

## The liquid biopsy

Tumors are heterogeneous and continuously evolve. Evolutionary changes within the cancer can alter the mutational spectrum of the disease and the responsiveness of the cancer to therapies. This may necessitate repeat biopsies [55]. However, repeat biopsies are difficult, invasive, costly and somewhat limited in that they provide a picture of mutations present only at a given time and organ site [55]. Recent studies have shown that genomic alterations in solid cancers can be characterized by massively parallel sequencing of circulating cell-free tumor DNA released from cancer cells into plasma, representing a non-invasive liquid biopsy. For some applications, mutation detection in plasma DNA could potentially replace invasive biopsies as a means to assess tumor genetic characteristics [55].

Genome-wide sequencing of plasma samples demonstrates comprehensive coverage of the genome [56]. More recently, genome-wide sequencing of plasma DNA has been demonstrated as a potential tool for detection of disease or analysis of tumor burden in patients with advanced cancers [55,57]. These studies have established that plasma DNA can represent the entire tumor genome [57]. However, identifying somatic mutations in cell-free tumor DNA is expensive and technically challenging.

Analysis of isolated circulating tumor cells (CTCs) has also been proposed. Characterization of CTCs could be used to predict treatment response and personalize cancer treatments [58]. However, isolation and characterization of CTCs is expensive and technically demanding [58]. Moreover, CTCs are not always present and may represent a different fraction of cells from the bulk metastatic tissue. While (serial) blood samples could be an interesting alternative to biopsies of metastatic lesions, we do not yet consider circulating DNA or CTCs as the solution to all our needs. Such an approach is useful for genomics, but does not inform on *in situ* transcriptome, proteome, lymphocytic infiltration or microenvironmental cues.

## Conclusion

The well-documented heterogeneity in the primary tumor supports the need for biopsy of one or more metastases. While biopsy of the metastatic site may not be prospectively able to demonstrate improved outcome for each patient - at least for the time being - it is paramount to introduce a culture of systematically biopsying metastases in order to gain information on the biology of the disease and, accordingly, select the best treatment option for our patients. This attitude is being introduced at present in clinical trials and may in future become standard clinical practice.

Hopefully, application of improved technology will ultimately translate into improved outcome. In this scenario, biomarker-driven clinical trials with targeted agents for patients with metastatic breast cancer with relatively rare but ‘actionable’ mutations may became crucial and biopsy of the metastatic tissues will be essential. We expect that future clinical trials will increasingly require metastatic tissue to be obtained to assess molecular differences, not only at the receptor level but also at the DNA, RNA, protein, and functional pathway levels. Most current target-driven therapeutic interventional trials will be developed according to a specific molecular profile.

The metastases could also be biopsied soon after initiation of treatment to determine by pharmacodynamic endpoints whether the disease is responding and whether treatment should be continued or discontinued or additional treatments should be added. At disease progression, biopsy of metastatic sites will be performed to elucidate the molecular changes contributing to treatment resistance, which may help to determine the next treatment choices.

Routine sampling of metastatic tissue will give us the opportunity to establish bio-banks of biopsy material taken from metastatic sites. Such biobanks will be important for exploring mechanisms of metastasis. In the future, advances in targeted therapy will depend on the availability of such metastatic tissue. We need to advance translational research by using analysis of metastatic tissue to design appropriate targeted therapy for patients with metastatic disease and ultimately for patient benefit.

## Abbreviations

BIG: Breast International Group; BRITS: Breast Recurrence In Tissues Study; CTC: Circulating tumor cell; ER: Estrogen receptor; HER: Human epidermal growth factor receptor; mTOR: Mammalian target of rapamycin; PgR: Progesterone receptor; PI3K: Phosphatidylinositide 3-kinase; PTEN: Phosphatase and tensin homolog.

## Competing interests

The authors declare that they have no competing interests.

## Authors’ contributions

CC and GC designed the study, collected and analyzed the data, and wrote the manuscript. FA, AMT, MDL, FO and AG read and critically revised the manuscript. All authors read and approved the final manuscript.
